# *ADAR1* polymorphisms are related to severity of liver fibrosis in HIV/HCV-coinfected patients

**DOI:** 10.1038/s41598-017-12885-4

**Published:** 2017-10-10

**Authors:** Luz M. Medrano, Juan Berenguer, María A. Jiménez-Sousa, Teresa Aldámiz-Echevarria, Francisco Tejerina, Cristina Diez, Lorena Vigón, Amanda Fernández-Rodríguez, Salvador Resino

**Affiliations:** 1 Unidad de Infección Viral e Inmunidad, Centro Nacional de Microbiología, Instituto de Salud Carlos III, Majadahonda, Madrid, Spain; 2 Unidad de Enfermedades Infecciosas/VIH, Hospital General Universitario “Gregorio Marañón”, Madrid, Spain; 3 Instituto de Investigación Sanitaria Gregorio Marañón (IiSGM), Madrid, Spain

## Abstract

The adenosine deaminase acting on RNA (*ADAR1*) gene is an interferon-stimulated gene involved in liver injury protection. Our aim was to analyze the association of polymorphisms within this gene with the severity of liver disease in European HIV/HCV-coinfected patients. We performed a cross-sectional study in 220 patients that underwent a liver biopsy. Five SNPs in the *ADAR1* gene (rs1127326, rs1127317, rs1127314, rs1127313, rs2229857) were genotyped by GoldenGate assay. The outcome variables were fibrosis stage and necroinflammatory activity grade by METAVIR-score, aspartate aminotransferase to platelet ratio index (APRI), FIB-4 index, and fibrosis progression rate (FPR). In multivariate analysis, fibrosis progression rate (FPR) (aAMRs = 0.97) decreased in a dose-dependent manner with the presence of rs2229857_T, rs1127313_G, rs1127314_G and rs1127317_G; while rs1127326_T allele had only significant associations with FIB-4 (aAMRs ≤ 0.63) and FPR (aAMRs ≤ 0.97). Moreover, carriers of rs2229857_T, rs1127314_G, rs1127317_G, and rs1127326_T alleles were protected against advanced fibrosis (F ≥ 3) (adjusted ORs (aORs) ≤ 0.44), APRI ≥ 1.5 (aORs ≤ 0.33), and FPR ≥ 0.075 (aORs ≤ 0.45). rs1127313_G carriers showed lower odds of having F ≥ 3 (aORs = 0.39), FIB4 ≥ 3.25 (aOR = 0.22) and FPR ≥ 0.075 (aORs = 0.44). In conclusion, *ADAR1* polymorphisms protected against severe liver disease in HIV/HCV-coinfected patients. These results could be used to improve therapeutic decision-making in clinical practice.

## Introduction

According to the World Health Organization, about 130–150 million people have chronic hepatitis C (CHC), but the actual number of infected people worldwide remains unknown^1^. Most hepatitis C virus (HCV) infections become persistent, and a significant number of chronically infected patients will develop severe liver disease resulting in cirrhosis or liver cancer^2^. This issue is a serious health burden as HCV causes 700,000 deaths each year from Hepatitis C-related liver diseases^3^.

The progression of liver disease takes place over several decades, but it is accelerated in the presence of various factors and comorbid conditions, such as coinfection with human immunodeficiency virus (HIV)^4^. Coinfection may influence the natural history of HCV, accelerating the progression to cirrhosis, liver decompensation, liver failure, and the development of hepatocellular carcinoma^5,6^.

Nowadays, new therapies have the ability to cure approximately 90% of HCV-infected patients^2^, but limited access to diagnosis and treatment in certain areas complicates HCV eradication efforts. Consequently, the assessment of liver disease severity is highly important in treatment selection and prognosis of patients in order to prioritize HCV treatment.

The progression of liver disease is highly variable, and the genetic background of the infected individual is an important factor^7^. Thus, the identification of single nucleotide polymorphisms (SNPs) as biological markers for liver disease is essential. Several SNPs in different genes have been found to be associated with liver-related diseases^8,9^, but most genetic variance still remains unknown.

During HCV infection, an array of interferon-stimulated genes (ISGs) are expressed to suppress viral replication, and we analyzed one of them to explore other sources of genetic variance.

The adenosine deaminase acting on RNA (*ADAR1*) gene is an ISG that is expressed during viral infection to suppress replication of RNA viruses by RNA editing. This effect is made by hydrolytic deamination of adenosines residues in dsRNA^10^, which leads to the conversion of adenosine to inosine and therefore destabilizes double-stranded RNA. Inosine residues are then transcribed and translated as guanosine, which may lead to mutations. This post-transcriptional modification is widely spread and highly conserved, which allows to regulate and diversify the transcriptome.

Three primary members of the ADAR family have been identified in humans: ADAR1, ADAR2 and ADAR3. ADAR1 is expressed in both the constitutive p110 isoform and the interferon-inducible p150 isoform. All of the ADAR enzymes contain a deaminase domain and differ in their Z-DNA-binding domains^11^, and they display significant differences in terms of regulation, protein domain structure, subcellular localization and biological function^12^. ADARs have been implicated in a wide range of human diseases such as cancer, neurological disorders, metabolic diseases, viral infections and autoimmune disorders^11^. In addition, the *ADAR1* gene has also shown regulatory functions in global miRNA biogenesis and RNA interference^13^, but its full biological significance remains elusive.

Only ADAR1, located in the nucleus, is an ISG induced by pathogen infections^14^. Expression and function of ADAR1 has been reported in different tissues, but intriguingly its deletion only generates morphological defects in the liver, suggesting a key role for maintaining adult liver homeostasis and its morphological and functional integrity^15,16^ while limiting the development of liver inflammation and fibrosis^15^. However, the mechanism by which ADAR1 protects the liver remains unknown^17,18^. Moreover, ADAR1 may display an antiviral activity against several RNA viruses, but other viruses, such as hepatitis delta virus^19^ and HIV^20^, utilize ADAR1 editing to promote the viral life cycle. The *ADAR1* gene is located on chromosome 1q21.3, and is comprised of 15 exons. To our knowledge, there is no information about the effect of *ADAR1* polymorphisms on CHC in patients coinfected with HIV and HCV. Therefore, we aimed to investigate the possible impact of genetic variants located in the *ADAR1* gene on liver disease severity in HIV/HCV-coinfected patients.

## Results

### Characteristics of the study population

The epidemiological and clinical characteristics of 220 HIV/HCV-coinfected patients at the time of liver biopsy are showed in Table 1. Results from the liver biopsy revealed that 22.3% had advanced fibrosis (F ≥ 3) and 10.5% had severe necroinflammatory activity (A ≥ 3).

**Table 1:** Clinical and epidemiological characteristics of HIV/HCV-coinfected patients.

Characteristics	All Patients
**No**.	220
**Male**	162 (73.6%)
**Age, years**	39.8 (37.4; 44.1)
**Epidemiological history**	
HIV acquired by IVDU	193 (87.7%)
Years since HCV infection	21.3 (17.1; 24.4)
High alcohol intake	120 (54.8%)
High alcohol intake at biopsy study	31 (14.2%)
CDC category C	60 (27.3%)
**Metabolic markers (n** = **218)**	
BMI, kg/m^2^	22.3 (20.8; 24.6)
BMI ≥ 25 kg/m^2^	50 (22.9%)
HOMA	2.09 (1.27; 3.73)
HOMA ≥ 3	71 (33.5%)
**Antiretroviral therapy**	
Time on cART, years	4.4 (2.5; 6.6)
**Current cART protocols**	
Non-treated	33 (15.0%)
PI-based	50 (22.7%)
NNRTI-based	114 (51.8%)
NRTI-based	23 (10.5%)
**HIV markers**	
Nadir CD4 + , T cells/μL	192 (84; 318)
CD4 + , T cells/μL	467 (324; 672)
HIV-RNA < 50 copies/mL	162 (73.6%)
**HCV markers**	
**HCV genotype (n = 216)**	
1	123 (56.9%)
2	5 (2.3%)
3	50 (23.1%)
4	38 (17.6%)
HCV RNA > 500,000 UI/mL (n = 217)	162 (74.7%)
**Liver biopsy (Metavir score)**	
**Hepatic fibrosis**	
F0	25 (11.4%)
F1	86 (39.1%)
F2	60 (27.4%)
F3	26 (11.8%)
F4	23 (10.5%)
**Activity grade (n = 215)**	
A1	102 (47.1%)
A2	90 (41.7%)
A3	23 (10.5%)
**Fibrosis indexes**	
APRI	0.75 (0.45–1.3)
APRI ≥ 1.5	36 (17.2%)
FIB-4	1.43 (1.03–2.04)
FIB-4 ≥ 3.25	20 (9.6%)

Values are expressed as absolute numbers (%) and median (percentile 25; percentile 75). Sometimes, the percentages were not calculated from all patients because some data was missing.

Abbreviations: BMI, body mass index; HOMA, homeostatic model assessment; HCV, Hepatitis C virus; CDC category C, centers for disease control and prevention classification system, clinical category C which includes any condition listed in the CDC’s 1897 surveillance case definition of AIDS; PI-based, protein inhibitor-based therapy; NNRTI-based, non-nucleoside reverse transcriptase inhibitor -based therapy; 3NRTI-based, triple nucleoside regimen; HIV; Human immunodeficiency virus; HIV-RNA, HIV plasma viral load; HCV-RNA, HCV plasma viral load.

### *ADAR1* polymorphisms

**Figure 1:**
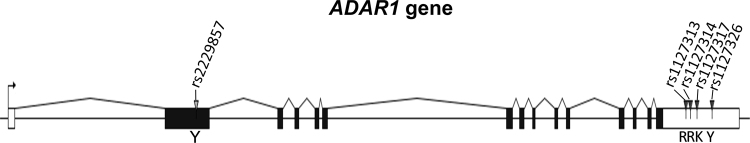
*ADAR1* gene with studied polymorphisms. Gray triangles show missense SNPs and black triangles 3′UTR SNPs. Nucleic acid substitution according to IUPAC Nucleic Acid Code: Y (C/T), R (A/C), and K (G/T). Alleles represented in forward strand. Figure designed with GenePalette software.

The *ADAR1* SNPs are located in exon 2 (rs2229857) and in the 3′ untranslated region (UTR) (rs1127313, rs1127314, rs1127317 and rs1127326) (Fig. 1).

The allelic and genotypic frequencies of the *ADAR1* polymorphisms are shown in Table 2. All SNPs were in Hardy-Weinberg equilibrium (HWE) (p > 0.05) and showed missing values <5%. The least common alleles were rs2229857 T (29.3%), rs1127313 G (49.1%), rs1127314 G (29.7%), rs1127317 G (29.1%), and rs1127326 T (29.5%). These frequencies were compared to those for healthy subjects extracted from the Iberian population in Spain (IBS) from the 1000 genomes database. We did not find any significant differences between groups.

**Table 2:** Summary of Hardy Weinberg Equilibrium and frequencies of alleles and genotypes for *ADAR1* polymorphisms in HIV/HCV-coinfected patients compared to Iberian populations in Spain from 1000 genomes project data (http://browser.1000genomes.org/index.html).

SNPs	HWE	HIV/HCV group (n = 220)				IBS group (n = 107)				χ² test(a)	χ² test (b)
	p-value	Alleles		Genotype		Alleles		Genotype		p-value	p-value
*ADAR1* rs2229857	0.745	C	70.7%	CC	50.5%	C	68.7%	CC	44.9%	0.602	0.342
		T	29.3%	CT	40.5%	T	31.3%	CT	47.7%	—	0.216
		—	—	TT	9.1%	—	—	TT	7.5%	—	0.624
*ADAR1* rs1127313	1.000	A	50.9%	AA	26.0%	A	51.9%	AA	25.2%	0.818	0.877
		G	49.1%	AG	49.8%	G	48.1%	AG	53.3%	—	0.552
		—	—	GG	24.2%	—	—	GG	21.5%	—	0.587
*ADAR1* rs1127314	0.871	A	70.3%	AA	49.8%	A	68.2%	AA	44.9%	0.584	0.404
		G	29.7%	AG	41.1%	G	31.8%	AG	46.7%	—	0.334
		—	—	GG	9.1%	—	—	GG	8.4%	—	0.829
*ADAR1* rs1127317	0.871	T	70.9%	TT	50.5%	T	68.7%	TT	44.9%	0.560	0.342
		G	29.1%	TG	40.9%	G	31.3%	TG	47.7%	—	0.247
		—	—	GG	8.6%	—	—	GG	7.5%	—	0.720
*ADAR1* rs1127326	0.743	C	70.5%	CC	50.2%	C	68.2%	CC	44.9%	0.559	0.363
		T	29.5%	TC	40.5%	T	31.8%	TC	46.7%	—	0.351
		—	—	TT	9.3%	—	—	TT	8.4%	—	0.792

Statistically significant differences are shown in bold. P-values were calculated by Chi-squared test; (a), differences between allele frequencies; (b), differences between genotype frequencies.

Abbreviations: HWE, Hardy Weinberg Equilibrium; HIV, human immunodeficiency virus; HCV, hepatitis C virus; IBS, Iberian populations in Spain; ADAR1, Double-stranded RNA-specific adenosine deaminase.

### *ADAR1* genotypes and liver disease

The additive model of inheritance was the model that best fit to our data. With this model, we explored the genetic association between *ADAR1* polymorphisms and liver disease stages (Tables 3–5).

**Table 3:** Relationship between *ADAR1* polymorphisms and continuous values of APRI, FIB-4, and FPR in HIV/HCV-coinfected patients.

	Unadjusted		Adjusted		
	AMR (95%CI)	p^(b)^	aAMR (95%CI)	p^(b)^	FDR
**rs2229857_TT**					
APRI	0.76 (0.60; 0.95)	**0.019**	0.79 (0.64; 0.98)	**0.032**	**0.060**
FIB-4	0.66 (0.47; 0.94)	**0.023**	0.68 (0.50; 0.92)	**0.013**	**0.021**
FPR	0.97 (0.95; 0.99)	**0.013**	0.97 (0.95; 0.99)	**0.027**	**0.034**
**rs1127313_GG**					
APRI	0.86 (0.70; 1.07)	**0.199**	0.81 (0.66; 0.99)	**0.048**	**0.060**
FIB-4	0.68 (0.49; 0.95)	**0.022**	0.63 (0.47; 0.81)	**0.002**	**0.010**
FPR	0.97 (0.95; 0.99)	**0.005**	0.97 (0.95; 0.99)	**0.001**	**0.005**
**rs1127314_GG**					
APRI	0.75 (0.60; 0.95)	**0.018**	0.79 (0.64; 0.97)	**0.032**	**0.060**
FIB-4	0.66 (0.47; 0.95)	**0.023**	0.68 (0.50; 0.92)	**0.013**	**0.021**
FPR	0.97 (0.95; 0.99)	**0.009**	0.97 (0.95; 0.99)	**0.022**	**0.034**
**rs1127317_GG**					
APRI	0.76 (0.60; 0.96)	**0.024**	0.80 (0.65; 0.99)	**0.047**	**0.060**
FIB-4	0.67 (0.47; 0.96))	**0.031**	0.69 (0.51; 0.95)	**0.021**	**0.021**
FPR	0.97 (0.95; 0.99)	**0.010**	0.97 (0.95; 0.99)	**0.027**	**0.034**
**rs1127326_TT**					
APRI	0.80 (0.65; 0.96)	**0.046**	0.82 (0.67; 1.01)	**0.060**	**0.060**
FIB-4	0.69 (0.48; 0.98)	**0.039**	0.69 (0.51; 0.94)	**0.017**	**0.021**
FPR	0.97 (0.95; 0.99)	**0.018**	0.97 (0.95; 0.99)	**0.039**	**0.039**

Statistically significant differences are shown in bold. ^(a)^P-values were calculated by univariate (a) and multivariate (b) generalized linear models (GLM) adjusted by the most important clinical and epidemiological characteristics (see statistical analysis section).

Abbreviations: 95%CI, 95% of confidence interval; AMR, arithmetic mean ratio; aAMR, adjusted arithmetic mean ratio; APRI, aspartate aminotransferase to platelet ratio index; FPR, fibrosis progression rate; ADAR1, Double-stranded RNA-specific adenosine deaminase; HCV, Hepatitis C virus; HIV, human immunodeficiency virus. FDR, False Discovery Rate.

**Table 4:** Relationship between *ADAR1* polymorphisms and ordinal values of liver fibrosis stage and activity grade in HIV/HCV-coinfected patients.

	Unadjusted		Adjusted		
	OR (95%CI)	p^(a)^	aOR (95%CI)	p^(b)^	FDR
**rs2229857_TT**					
Fibrosis Stage	0.66 (0.46; 0.96)	**0.030**	0.64(0.42; 0.97)	**0.035**	**0.035**
Activity Grade	0.98 (0.66; 1.43)	0.909	0.99 (0.64; 1.54)	0.980	0.980
**rs1127313_GG**					
Fibrosis Stage	0.68 (0.48; 0.97)	**0.033**	0.54(0.35; 0.81)	**0.003**	**0.015**
Activity Grade	1.20 (0.84; 1.70)	0.304	1.11 (0.74; 1.68)	0.605	0.605
**rs1127314_GG**					
Fibrosis Stage	0.65 (0.45; 0.94)	**0.022**	0.62 (0.41; 0.94)	**0.024**	**0.035**
Activity Grade	0.98 (0.66; 1.44)	0.917	0.98 (0.63; 1.53)	0.951	0.951
**rs1127317_GG**					
Fibrosis Stage	0.65 (0.45; 0.95)	**0.025**	0.63 (0.41; 0.95)	**0.030**	**0.035**
Activity Grade	0.93 (0.63; 1.37)	0.721	0.94 (0.60; 1.46)	0.776	0.776
**rs1127326_TT**					
Fibrosis Stage	0.67 (0.46; 0.97)	**0.034**	0.63 (0.42; 0.96)	**0.034**	**0.035**
Activity Grade	0.97 (0.66; 1.42)	0.888	0.97 (0.62; 1.51)	0.897	0.897

Statistically significant differences are shown in bold. ^(a)^p-values were calculated by univariate ordinal regression; ^(b)^p-values were calculated by multivariate ordinal regression adjusted by the most important clinical and epidemiological characteristics (see statistical analysis section).

Abbreviations: 95%CI, 95% of confidence interval; OR, odds ratio; aOR, adjusted odds ratio; ADAR1, Double-stranded RNA-specific adenosine deaminase; HCV, Hepatitis C virus; HIV, human immunodeficiency virus. FDR, False Discovery Rate.

**Table 5:** Summary of the relationship between *ADAR1* polymorphisms and severity of liver disease in HIV/HCV coinfected patients.

*ADAR1* rs2229857	CC	TC	TT	p-value^(a)^	aOR (95%CI)	p-value^(b)^	FDR
F ≥ 3	29.7% (33/111)	15.7% (14/89)	10.0% (2/20)	**0.008**	0.43 (0.22; 0.82)	**0.011**	**0.015**
A3	15.7% (17/108)	7.9% (7/89)	0.0% (0/19)	**0.018**	0.38 (0.13; 1.08)	0.070	0.098
APRI ≥ 1.5	24.0% (25/104)	11.6% (10/86)	5.3% (1/19)	**0.009**	0.32(0.14; 0.72)	**0.007**	**0.012**
FIB4 ≥ 3.25	13.5% (14/104)	5.8% (5/86)	5.3% (1/19)	0.079	0.38 (0.14; 1.07)	0.068	0.095
FPR ≥ 0.075	58.4% (59/101)	40.5% (34/84)	42.1% (8/19)	**0.026**	0.44 (0.26; 0.78)	**0.004**	**0.005**
***ADAR1*** **rs1127313**	**AA**	**AG**	**GG**	**p-value** ^**(a)**^	**aOR (95%CI)**	**p-value** ^**(b)**^	
F ≥ 3	29.8% (17/57)	23.9% (26/109)	9.4% (5/53)	**0.011**	0.39 (0.21; 0.71)	**0.002**	**0.010**
A3	10.5% (6/57)	14.2% (15/106)	5.8% (3/52)	0.458	0.74 (0.32; 1.68)	0.470	0.470
APRI ≥ 1.5	18.9% (10/53)	19.2% (20/104)	11.8% (6/51)	0.345	0.49 (0.24; 1.02)	0.057	0.057
FIB4 ≥ 3.25	13.2% (7/53)	10.6% (11/104)	3.9% (2/51)	0.111	0.22 (0.07; 0.68)	0.008	**0.040**
FPR ≥ 0.075	55.8% (29/52)	52% (52/100)	37.3% (19/51)	0.062	0.44 (0.27; 0.74)	0.002	**0.010**
***ADAR1*** **rs1127314**	**AA**	**AG**	**GG**	**p-value** ^**(a)**^	**aOR (95%CI)**	**p-value** ^**(b)**^	
F ≥ 3	30.3% (33/109)	15.6% (14/90)	10.0% (2/20)	**0.006**	0.41 (0.21; 0.80)	0.009	0.015
A3	15.1% (16/106)	7.8% (7/90)	0.0% (0/19)	**0.023**	0.38 (0.13; 1.10)	0.074	0.098
APRI ≥ 1.5	24.5% (25/102)	11.5% (10/87)	5.3% (1/19)	**0.007**	0.31 (0.13; 0.70)	**0.005**	**0.012**
FIB4 ≥ 3.25	13.7% (14/102)	5.7% (5/87)	5.3% (1/19)	0.070	0.36 (0.13; 1.04)	0.059	0.095
FPR ≥ 0.075	58.6% (58/99)	40% (34/85)	42.1% (8/19)	**0.023**	0.44 (0.25; 0.77)	**0.004**	**0.005**
***ADAR1*** **rs1127317**	**TT**	**TG**	**GG**	**p-value** ^**(a)**^	**aOR (95%CI)**	**p-value** ^**(b)**^	
F ≥ 3	29.7% (33/111)	15.6% (14/90)	10.5% (2/19)	**0.008**	0.43 (0.22;0.83)	**0.012**	**0.015**
A3	15.7% (17/108)	7.8% (7/90)	0.0% (0/18)	**0.018**	0.39 (0.13; 1.11)	0.078	0.098
APRI ≥ 1.5	24.0% (25/104)	11.5% (10/87)	5.6% (1/18)	**0.009**	0.31(0.13; 0.73)	**0.007**	**0.012**
FIB4 ≥ 3.25	13.5% (14/104)	5.7% (5/87)	5.6% (1/18)	0.082	0.38 (0.13; 1.10)	0.076	0.095
FPR ≥ 0.075	58.4% (59/101)	41.2% (35/85)	38.9% (7/18)	**0.018**	0.43 (0.25; 0.76)	**0.004**	**0.005**
***ADAR1*** **rs1127326**	**CC**	**CT**	**TT**	**p-value** ^**(a)**^	**aOR (95%CI)**	**p-value** ^**(b)**^	
F ≥ 3	28.7% (31/108)	16.1% (14/87)	10.0% (2/20)	**0.014**	0.44 (0.23; 0.85)	**0.015**	**0.015**
A3	16.2% (17/105)	8% (7/87)	0.0% (0/19)	**0.016**	0.36 (0.12; 1.05)	0.061	0.098
APRI ≥ 1.5	22.8% (23/101)	11.8% (10/85)	5.3% (1/19)	**0.016**	0.33(0.15; 0.77)	**0.010**	**0.013**
FIB4 ≥ 3.25	11.9% (12/101)	5.9% (5/85)	5.3% (1/19)	0.151	0.43 (0.15; 1.24)	0.118	0.118
FPR ≥ 0.075	57% (57/98)	33% (33/82)	8% (8/19)	**0.030**	0.45 (0.26; 0.79)	**0.005**	**0.005**

Statistically significant differences are shown in bold. ^(a)^P-values were calculated by Extended Mantel Haenszel Chi Square for linear trend; ^(b)^P-values were calculated by multivariate logistic regression adjusted by the most important clinical and epidemiological characteristics (see statistical analysis section).

Abbreviations: 95%CI, 95% of confidence interval; aOR, adjusted odds ratio; p-value, level of significance; F ≥ 3, advanced fibrosis(Metavir); A3, severe activity grade (Metavir); APRI, aspartate aminotransferase to platelet ratio index; FPR, fibrosis progression rate; ADAR1, Double-stranded RNA-specific adenosine deaminase; HCV, Hepatitis C virus; HIV, human immunodeficiency virus. FDR, False Discovery Rate.

Firstly, the association values with continuous variables are shown in Table 3. In multivariate analysis, the values of FIB-4 (aAMRs ≤ 0.69; p < 0.05), and fibrosis progression rate (FPR) (aAMRs = 0.97; p < 0.05) decreased in a dose-dependent manner with the presence of rs2229857 T, rs1127313 G, rs1127314 G and rs1127317 G; while the rs1127326 T allele had only significant values for FIB-4 (aAMRs = 0.69; p < 0.05) and FPR (aAMRs = 0.97; p < 0.05).

Secondly, the association values with ordinal variables are shown in Table 4. Patients carrying the rs2229857 T, rs1127313 G, rs1127314 G, rs1127317 G, and rs1127326 T alleles were protected against the increase of fibrosis stage in the multivariate analysis [adjusted ORs (aORs) ≤ 0.64; p < 0.05]. No significant values were found for the activity grade.

Next, the association values with dichotomous variables are shown in Table 5. In the multivariate analysis, carriers of rs2229857 T, rs1127314 G, rs1127317 G, and rs1127326 T alleles were protected against advanced liver fibrosis (F ≥ 3) (aORs ≤ 0.44; p < 0.05), values of APRI ≥ 1.5 (aORs ≤ 0.33; p < 0.05), and FPR ≥ 0.075 (aORs ≤ 0.45; p < 0.05). In the same way, patients carrying the rs1127313 G allele showed lower odds of having advanced liver fibrosis (F ≥ 3) (aORs = 0.39; p = 0.002), values of FIB4 ≥ 3.25 (aOR = 0.22; p = 0.008) and FPR ≥ 0.075 (aORs = 0.44; p = 0.002). No significant values were found for the activity grade.

### *ADAR1* haplotypes and liver disease

**Figure 2:**
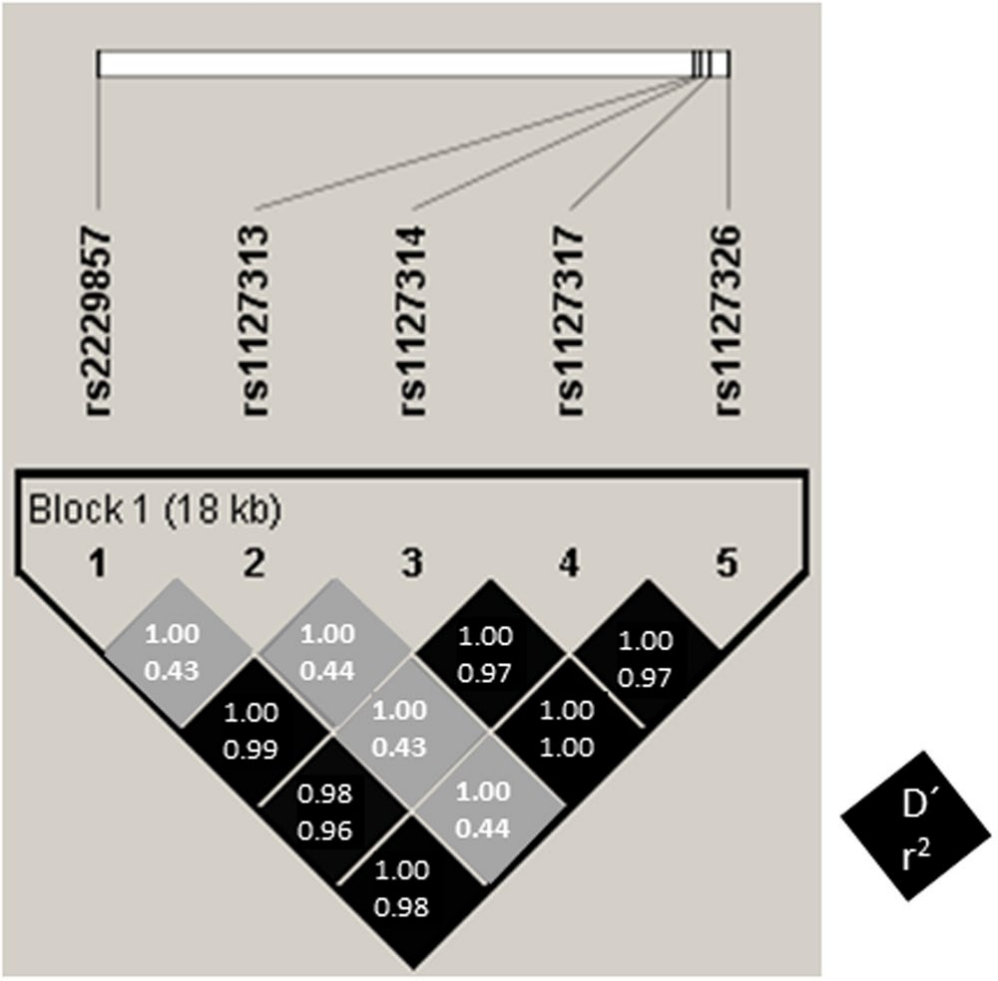
Pairwise linkage disequilibrium (LD) pattern for *ADAR1* polymorphisms. Each diagonal represents a different SNP, with each square representing a pairwise comparison between two SNPs.

Linkage disequilibrium (LD) analysis (Fig. 2) showed that there was a high LD (non-random association of alleles at different loci) between *ADAR1* SNPs (D’ > 0.98), meaning that there is no evidence of a possible recombination between these SNPs. Moreover, the R-squared among all studied SNPs, except for rs1127326, was high (R^2^ > 0.96). R^2^ values among all SNPs and rs1127313 is less than 0.44, meaning that rs1127313 did not provide the same information and could not be substituted one for the other. Furthermore, we performed a Tag SNPs analysis in the region studied by using Haploview 4.2 software and we determined that rs1127313 and rs1127317 are representative SNPs in our study.

Haplotype analysis (Table 6) showed that only three major *ADAR1* haplotypes (comprised of rs1127313 and rs1127317) were found: 51% AT (unfavorable alleles), 28.8% GG (favorable alleles), and 19.4% GT. The AT haplotype (unfavorable alleles) showed higher odds for having advanced liver fibrosis (F ≥ 3) (aOR = 2.63; p = 0.006), values of FIB4 ≥ 3.25 (aOR = 2.26; p = 0.008) and rapid fibrosis progression (FPR ≥ 0.075) (aOR = 2.27; p = 0.024); whereas the GG haplotype (favorable alleles) showed lower odds for having advanced liver fibrosis (F ≥ 3) (aOR = 0.44; p = 0.022), values of APRI ≥ 1.5 (aOR = 0.33; p = 0.027), and values of FPR ≥ 0.075 (aOR = 0.45; p = 0.007).

**Table 6:** Haplotype frequencies of *ADAR1* rs1127313 and rs1127317 polymorphisms, and their genetic association with the severity of liver disease in HIV/HCV-coinfected patients on HCV therapy.

	Frequency		OR (95%CI)	p-value^(a)^	^a^OR (95%CI)	p-value^(b)^	FDR
**Haplotypes by fibrosis stage**	**F < 3**	**F ≥ 3**					
AT	47.7	62.7	1.86 (1.16; 2.99)	**0.010**	2.63 (1.43; 4.83)	**0.002**	**0.006**
GG	31.9	18.4	0.48 (0.27; 0.84)	**0.011**	0.44 (0.22; 0.84)	**0.015**	**0.022**
GT	19.6	18.9	0.95 (0.52; 1.74)	0.878	0.74 (0.36; 1.52)	0.415	0.415
**Haplotypes by activity grade**	**A < 3**	**A3**					
AT	50.6	56.2	1.25 (0.69; 2.28)	0.467	1.33 (0.58; 3.02)	0.498	0.498
GG	30.7	14.6	0.38 (0.16; 0.88)	**0.024**	0.39 (0.14; 1.13)	0.082	0.246
GT	17.9	29.2	2.06 (0.99; 4.28)	0.054	1.98 (0.74; 5.27)	0.173	0.259
**Haplotypes by APRI**	**APRI < 1.5**	**APRI ≥ 1.5**					
AT	49.6	55.6	1.27 (0.76; 2.13)	0.354	2.01 (0.98; 4.15)	0.058	0.087
GG	31.8	16.7	0.42 (0.21; 0.83)	**0.012**	0.33 (0.14; 0.76)	**0.009**	**0.027**
GT	17.8	27.8	1.92 (1.02; 3.61)	**0.044**	1.56 (0.73; 3.39)	0. 251	0.251
**Haplotypes by FIB4**	**FIB4** ** < 3.25**	**FIB4** ** ≥ 3.25**					
AT	49.3	62.5	1.47 (0.99; 2.17)	0.117	2.26 (1.36; 3.79)	**0.008**	**0.024**
GG	30.4	17.5	0.61(0.39; 0.94)	0.094	0.45(0.25; 0.78)	0.087	0.13
GT	19.5	20.0	1.08 (0.65; 1.81)	0.931	0.84 (0.45; 1.59)	0.428	0.42
**Haplotypes by FPR**	**FPR < 0.075**	**FPR ≥ 0.075**					
AT	45.6	55.2	1.46 (0.98; 2.17)	0.057	2.27 (1.36; 3.79)	**0.002**	**0.006**
GG	34.5	24.3	0.61 (0.39; 0.94)	**0.026**	0.45 (0.26; 0.78)	**0.005**	**0.007**
GT	18.9	20.1	1.08 (0.64; 1.81)	0.760	0.84 (0.45; 1.59)	0.594	0.594

Statistically significant differences are shown in bold. (a) P-values were calculated by logistic regression model. (b) P-values were calculated by logistic regression model adjusting for the most important clinical and epidemiological characteristics (see statistical analysis section).

Abbreviations: OR, odds ratio; aOR, adjusted odds ratio; 95%CI, 95% confidence interval; F ≥ 3, advanced fibrosis(Metavir); A3, severe activity grade (Metavir); APRI, aspartate aminotransferase to platelet ratio index; FPR, fibrosis progression rate; ADAR1, Double-stranded RNA-specific adenosine deaminase; HCV, Hepatitis C virus; HIV, human immunodeficiency virus. FDR, False Discovery Rate.

### *In silico* analysis

The *in silico* prediction analysis (Supplementary Tables 1–4) showed functional predictions in all the markers studied. rs2229857 is a missense variant located in exon 2 and produces an amino acid substitution (K/R), which does not seem to affect the protein structure according to SIFT and PolyPhen tools. Furthermore, according to the F-SNP database, rs2229857 seems to alter a putative exonic splicing enhancer (ESE). Functional analysis of rs2229857, rs1127313, rs1127314, rs1127317 and rs1127326 polymorphisms by using rSNPBase reported that each of them could be involved in transcriptional regulation. Additionally, rs1127313 and rs1127314 are 3′UTR variants and seem to be involved in the miRNA binding site of hsa-miR-4516 and hsa-miR-363-5p, respectively.

## Discussion

In this study, the major findings were that *ADAR1* rs2229857 T, rs1127313 G, rs1127314 G, rs1127317 G, and rs1127326 T alleles protect against the development of liver fibrosis, (fibrosis stage, but not activity grade) and the development of high levels of non-invasive indexes (APRI and FIB-4) in HIV/HCV-coinfected patients. We have detected these effects both with *ADAR1* SNPs alone and *ADAR1* haplotypes; similar associations were observed in both analyses. To the best of our knowledge, this is the first study showing a genetic association between *ADAR1* SNPs and the severity of liver fibrosis in HIV/HCV-coinfected patients.

The immune response against HCV infection initially attempts to eradicate the viral infection, but the immune system also causes hepatocyte damage and fibrosis through direct cellular toxicity and the release of inflammatory cytokines^21^. The innate response against viruses produces type I IFN (alpha, beta), which induces the expression of ISGs such as ADAR1^22^. In fact, a high level of ADAR1 protein has been detected in HCV-infected patients at weeks 4–12 during PEG-IFN alfa-2a plus ribavirin (IFN2a/r)^23^ therapy. Additionally, the inhibition of ADAR1 *in vitro* stimulates the production of an HCV-replicon, indicating that ADAR1 has a role in limiting HCV replication^18^. Moreover, *ADAR1* SNPs have been related to HCV clearance in Caucasian American patients treated with IFN2a/r^24^, and HBV clearance in Chinese patients, both spontaneously and with interferon treatment^25^.

HCV-related liver fibrosis is a progressive disease that results from an impaired immune response and subsequent loss of control of HCV replication^7^. The lack of induction of an ISG-mediated antiviral response has been associated with rapid fibrosis progression in HCV infected and HCV/HIV coinfected patients^26^. Additionally, the *ADAR1* gene seems to contribute to the regulation of innate immunity, especially in hepatocytes^16^. Thus, ADAR1 may have implications in liver diseases involving excessive inflammation that may induce liver fibrosis in HCV-infected patients^18^. Therefore, any genetic variant at *ADAR1* gene that impairs its protein expression or stability, could be affecting to liver fibrosis progression. In addition, recent reports have shown that the *ADAR1* gene is also related to HIV infection, as ADAR1 protein seems to be incorporated into HIV virions^20^, where it is required for efficient replication in CD4+ T cells^27^.

With respect to the functionality of the *ADAR1* SNPs, we have performed an *in silico* prediction of the putative role of each *ADAR1* polymorphism on protein function or expression. The rs2229857A > G is a missense polymorphism that generates a lysine to arginine amino acid change at position 427 in the protein. This change could affect the solvent accessibility of the protein and the transcriptional regulation^28^. The *in silico* prediction showed that rs2229857T > C seems to alter a putative exonic splicing enhancer (ESE), which is an element that plays an important role in constitutive and alternative splicing^28^. Therefore, this variant could alter not only the structure of the protein, but also the RNA sequence and secondary structure. Furthermore, the functional analysis of rs1127313, rs1127314, rs1127317 and rs1127326 polymorphisms reported that each one could be involved in transcriptional regulation, being putative binding sites for different transcription factors.

ADAR proteins also play an important role in microRNA (miRNA) biogenesis. miRNAs are small non-coding cellular RNAs that are involved in post-transcriptional regulation of messenger RNAs (mRNAs). The mRNA is degraded or not transcribed depending on the complementarity between the miRNA and the target sequence of the mRNA. ADAR proteins act on nuclear double-stranded RNA structures that are pre-mRNA transcripts. Therefore, it is likely that they edit pri-miRNA transcripts prior to their export from the nucleus. In addition, ADAR1 is known to form a complex with Dicer^13^, an RNAse III enzyme involved in miRNA maturation.

All the studied SNPs, except for rs1127326, have been related to miRNA biogenesis^29^. Thus, the presence of polymorphisms in miRNA processing machinery genes such as *ADAR1* has an unpredictable and broad effect on transcriptional regulation. In addition, the presence of polymorphisms within the target sequence of the mRNA will affect miRNA recognition and therefore, the transcriptional regulation of the mRNA will be altered. In this respect, rs1127313 and rs1127314 are in the target sequence for the binding of hsa-miR-4516 and hsa-miR-363-5p, respectively. When the G allele for both SNPs is present, these miRNAs can bind to the ADAR1 mRNA and thus regulate its expression, while when the alternative allele (T) is present, the target is disrupted and the miRNA cannot bind to it. In our study, the presence of G alleles at both polymorphisms (rs1127313 and rs1127314), alone or in haplotype analysis, were associated with advanced liver fibrosis. On the one hand, hsa-miR-4516 seems to be involved in fibrosis reduction in muscle^30^. On the other hand, hsa-miR-363-5p has been identified as being upregulated in cirrhotic livers^31^, therefore its binding to ADAR1 could be related to a poor prognosis of liver disease.

The strong association between *ADAR1* SNPs and the severity of liver fibrosis has not been previously found in two previously performed genome-wide association studies (GWAS)^32,33^. This issue could be due to the fact that a GWAS usually does not have the power to detect all significant effects, only the biggest ones. In the GWAS reported^32,33^, the patients origins are not stated, therefore the lack of detection could be due to a different genetic background of the patients enrolled. In addition, both GWAS were performed with HCV patients, while our CHC patients were coinfected with HIV. In this case, the HIV could be affecting the relationship between the *ADAR1* polymorphisms and liver fibrosis development.

Finally, several aspects must be considered for the correct interpretation of the results. Firstly, this report has a cross-sectional design with a relatively small number of patients, which could limit the statistical power to detect some differences between groups. Therefore, it would be necessary to perform an independent replication of this study in a prospective study with a higher sample size, including different ethnic groups. Also, data was collected retrospectively, which entails a lack of uniformity. Secondly, although we found a strong association with *ADAR1* polymorphisms and liver fibrosis, we cannot assure that any of them are the causal mutation. Therefore, it cannot be discarded that the detected association is due to other SNPs in high linked disequilibrium with *ADAR1* polymorphisms. Thirdly, as discussed above, the *in silico* analysis showed that *ADAR1* polymorphisms alone could have some influence on the expression of the proteins involved, but it would be necessary to demonstrate if these *ADAR1* polymorphisms modify the expression of the *ADAR1* gene. Fourthly, all selected patients met a set of criteria for starting HCV treatment (e.g., no alcohol abuse, high CD4 cell counts, controlled HIV replication, and good treatment adherence), and this may have introduced a selection bias. Fifthly, this study was performed on patients with European ancestry, and it would be interesting to perform these analyses on different ethnic groups. Finally, our study only included HIV/HCV-coinfected patients and it would be interesting to know the role of the studied polymorphisms in HCV-monoinfected patients, but we did not have access to a cohort of HCV-monoinfected patients.

In conclusion, genetic variants in the *ADAR1* gene protect against the development of liver fibrosis in HIV/HCV-coinfected patients. These data suggest that *ADAR1* polymorphisms may play a major role in the pathogenesis of CHC in HIV/HCV-coinfected patients, and it could be used to improve therapeutic decision-making in clinical practice.

## Materials and Methods

### Study population

We performed a cross-sectional study in 220 HIV/HCV-coinfected naïve patients that underwent a liver biopsy between September 2000 and November 2008. The study was conducted in accordance with the Declaration of Helsinki, and patients gave their informed consent for the study. The Institutional Review Board and the Research Ethics Committee of the Instituto de Salud Carlos III approved the study.

Liver biopsies were performed on patients who were potential candidates for anti-HCV therapy and had not received previous IFN therapy (naïve for HCV-treatment). Selection criteria were: no clinical evidence of hepatic decompensation, detectable HCV RNA by polymerase chain reaction (PCR), negative hepatitis B surface antigen, availability of DNA sample, CD4 + lymphocyte count higher than 200 cells/µL, and stable combination antiretroviral therapy (cART) for at least 6 months before study entry or no need for cART according to treatment guidelines used in the study period^34,35^. Patients with active opportunistic infections, active drug addiction, and other concomitant severe diseases were excluded. All subjects included in our study were white Europeans.

### Epidemiological and clinical data

Clinical and epidemiological data were obtained from medical records.

Consumption of more than 50 g of alcohol per day for at least 12 months was considered as a high intake. Body mass index (BMI) was calculated as the weight in kilograms divided by the square of the height in meters. The duration of HCV infection for patients with a history of intravenous drug use (IDU; 87.7% of patients) was estimated starting from the first year they shared needles and other injection paraphernalia, which are the most relevant risk practices for HCV transmission^36^. When the initiation of HCV infection could not be estimated with certainty, the duration was not calculated (n = 16). For 204 out of 220 patients with the duration of HCV infection, the FPR was calculated by dividing the fibrosis stage (0 to 4) by the estimated duration of HCV infection in years (fibrosis units per year)^37^.

Biochemistry panel was measured using an autoanalyzer Hitachi 912 (Boehringer Mannheim, Germany), while patients were fasting. The degree of insulin resistance was estimated for each patient using the homeostatic model assessment (HOMA)^38^: fasting plasma glucose (mmol/L) times fasting serum insulin (mU/L) divided by 22.5. Non-invasive fibrosis indexes were calculated according to the formula originally described for the APRI^39^: aspartate aminotransferase [[AST. (/U/L)]/platelet [10^9^/L]] × 100 and the FIB-4 index^40^: age [years] × AST [U/L])/((platelets [10^9^/L]) × (alanine aminotransferase [ALT. U/L])^1/2^).

### HCV assays

HCV infection was documented in all patients by enzyme-linked immunosorbent assay and PCR test. HCV genotype was determined by hybridization of biotin-labeled PCR products to oligonucleotide probes bound to nitrocellulose membrane strips (INNO-LiPA HCV II, Innogenetics, Ghent, Belgium). Plasma HCV-RNA viral load was measured by PCR (Cobas Amplicor HCV Monitor Test, Branchburg, NJ, USA) and real-time PCR (COBAS AmpliPrep/COBAS TaqMan HCV test), and results were reported in terms of international units per milliliter (IU/mL). The limit of detection varied between 10 IU/mL and 600 IU/mL depending on the HCV diagnostic test used at the time of biopsy. There was no patient with HCV viral load below the limit of detection. The HCV RNA level of 500,000 UI/ml was chosen as cut-off for low and high viral load (circulating HCV RNA)^41,42^.

### Liver biopsy

Liver biopsies were performed as outpatient service following the recommendations of the Patient Care Committee of the American Gastroenterological Association^43^, as we have previously described^44^. All samples were evaluated by the same pathologist, who was unaware of the patients’ clinical or laboratory data. Liver fibrosis and necroinflammatory activity were estimated according to Metavir score as follows^45^: F0, no fibrosis; F1, mild fibrosis; F2, significant fibrosis; F3, advanced fibrosis; and F4, definite cirrhosis. The degree of necroinflammation (activity grade) was scored as follows: A0, no activity; A1, mild activity; A2, moderate activity; A3, severe activity.

### Genotyping of DNA polymorphisms

The most common SNPs in the *ADAR1* gene (chromosome 1) were selected using the databases of the HapMap Project (http://snp.cshl.org/cgi-perl/gbrowse/hapmap_B35/) and NCBI (dbSNP) (http://www.ncbi.nlm.nih.gov/entrez/). The selection criteria were: i) SNPs located in putative regulatory regions, or splicing control elements (SCE), or missense variants; ii) minor allelic frequency (MAF) greater than 20% in European people in order to have guarantees to obtain significant associations when working with small sample sizes^46^. With this setting, we found five SNPs in the *ADAR1* gene (rs1127326, rs1127317, rs1127314 and rs1127313 located at 3′UTR; and rs2229857 in exon 2)

Genomic DNA was extracted from peripheral blood with Qiagen kit (QIAamp DNA Blood Midi/Maxi; Qiagen, Hilden, Germany). DNA samples were genotyped at the Spanish National Genotyping Center (CeGen; http://www.cegen.org/) by using GoldenGate assay with VeraCode Technology (Illumina Inc., San Diego, California, USA).

We obtained the allelic and genotypic frequency for healthy subjects, from the 1000 Genomes Project website (http://www.1000genomes.org/home). This database provides a broad representation of common human genetic variation from multiple populations^47^. We selected the IBS population that included 107 individuals.

### Outcome variables

HIV/HCV-coinfected patients were classified according to the severity of liver disease developed after a minimum follow-up time of 10 years with HCV infection. The outcome variables were the fibrosis stage and the necroinflammatory activity grade according to METAVIR score, and values of APRI, FIB-4 and FPR. The cut-off values to determine the severity of liver disease were^48^: a) advanced fibrosis (F ≥ 3, APRI ≥ 1.5, and FIB-4 ≥ 3.25), b) severe activity grade (A3); and c) rapid fibrosis progression rate (FPR) higher than median (FPR ≥ 0.075).

### Statistical analysis

For the description of the study population, values were shown as absolute numbers (%) and median (percentile 25; percentile 75). Frequencies of alleles and genotypes for *ADAR1* SNPs in HIV/HCV-coinfected patients were compared to healthy subjects by using Chi-squared test.

Hardy-Weinberg equilibrium (HWE) for all SNPs was assessed by a χ^2^ test, considering equilibrium when p > 0.05. In addition, pair-wise linkage disequilibrium (LD) analysis was computed to detect the inter-marker relationship using the standardized D’ and r^2^ values by Haploview 4.2 software. The haplotype frequencies were inferred with the Expectation– maximization algorithm by using Haploview 4.2 software (https://www.broadinstitute.org/haploview/haploview). Haplotype association tests are obtained by summing the fractional likelihoods of each patient for each haplotype. The haplotype-based association test was performed by multivariate logistic regression adjusted by the most important clinical and epidemiological covariates, as cited above. These analyses were performed with Plink software (http://zzz.bwh.harvard.edu/plink/) and adjusted by the same variables as for association with individual SNPs. All p-values were two-tailed and statistical significance was defined as p < 0.05.

For the genetic association study, Generalized Linear Models (GLMs) were used to study the association of *ADAR1* polymorphisms with outcome variables according to different models of inheritance: dominant, recessive and additive. We used the Akaike information criterion (AIC) value and Bayesian information criterion (BIC) to evaluate the model that best fit our data. On the one hand, a GLM with a normal distribution was used to investigate the association between *ADAR1* polymorphisms and continuous outcome variables (APRI, FIB-4, and FPR). This test gives the differences between groups and the arithmetic mean ratio (AMR) in continuous outcome variables. On the other hand, a GLM with multinomial distribution (cumlogit-link) was used to investigate the association of *ADAR1* polymorphisms with ordinal outcome variables (fibrosis stage and activity grade). Next, a GLM with binomial distribution (logit-link) was used to investigate the association of *ADAR1* polymorphisms with categorical outcome variables (F ≥ 3, A3, APRI ≥ 1.5, FIB4 ≥ 3.25, and FPR ≥ 0.075). These tests give the differences between groups and the odds ratio (OR) for categorical outcome variables. Additionally, GLM tests were adjusted by the most important clinical and epidemiological characteristics (gender, age, alcohol intake, BMI, HOMA, nadir CD4+ T-cells, AIDS, undetectable HIV-RNA (<50 copies/ml), CD4+ T-cells, time on cART, type of cART, HCV-RNA ≥ 500,000 IU/ml, HCV genotype, *IL28B* rs12980275 polymorphism). All analyses were performed by using the IBM SPSS Statistics for Windows, Version 21.0 (IBM Corp, Chicago, Armonk, NY, USA).

All association analyses were corrected by multiple comparison testing by using the SDM software (http://www.sdmproject.com/utilities/?show=FDR) that calculate the False Discovery Rate (FDR)^49^.

We also performed an *in silico* analysis, for evaluating the possible functional implication of each polymorphism, by using different web-tools: a) F-SNP (http://compbio.cs.queensu.ca/F-SNP/) that provides information about the functional effects of SNPs obtained from 16 bioinformatics tools and databases^28^ b) Ensembl’s Variant Effect Predictor (VEP) tool (http://grch37.ensembl.org/info/docs/tools/vep/index.html)^50^ c) rSNPBase (http://rsnp.psych.ac.cn/) that provides regulatory annotations on rSNPs; and d) miRDB target prediction (http://mirdb.org/miRDB/index.html) for identification of miRNA target sequences^51,52^.

### Meetings at which parts of the data will be presented

Some parts were presented in the *VIII Conference GESIDA (GESIDA 2016)*, held in San Sebastian, Spain in November 29- December 2^nd^, 2016 and in the annual Conference on Retroviruses and Opportunistic Infections (CROI 2017), held in Seattle, USA on 13–16 February, 2017.

## Electronic supplementary material

Supplementary tables 1-4
