# Supplementary material for: ADAR1 polymorphisms are related to severity of liver fibrosis in HIV/HCV-coinfected patients
Source: Sci Rep. 2017 Oct 10;7:12918. doi: 10.1038/s41598-017-12885-4 (PMC5635123; doi:10.1038/s41598-017-12885-4)
Supplement: Supplementary file 1 — Supplementary tables 1-4 [file 41598_2017_12885_MOESM1_ESM.doc]

**Supplementary information**

*ADAR1* polymorphisms are related to severity of liver fibrosis in HIV/HCV-coinfected patients

Luz M MEDRANO; Juan BERENGUER; María A JIMÉNEZ-SOUSA; Teresa ALDÁMIZ-ECHEVARRIA; Francisco TEJERINA; Cristina DIEZ; Lorena VIGÓN; Amanda FERNÁNDEZ-RODRÍGUEZ ^(*)^; Salvador RESINO ^(*)^.

^(*)^contributed equally to this work.

**Supplementary table 1.** Summary of *in silico* functional predictions of rs2229857, rs1127313, rs1127314, rs1127317 and rs1127326 SNPs located within the *ADAR1* gene by using rSNPBase (<http://rsnp.psych.ac.cn/>)

|  | SNPs | rSNP | LD-proxy of rSNP | Proximal regulation | Distal regulation | miRNA regulation | RNA binding protein mediated regulation | eQTL |
| --- | --- | --- | --- | --- | --- | --- | --- | --- |
| rSNPBase | rs2229857 | Yes | Yes | No | Yes | No | Yes | Yes |
|  | rs1127313 | Yes | Yes | Yes | Yes | No | Yes | Yes |
|  | rs1127314 | Yes | Yes | Yes | Yes | No | Yes | Yes |
|  | rs1127317 | Yes | Yes | Yes | Yes | No | Yes | Yes |
|  | rs1127326 | Yes | Yes | Yes | Yes | No | Yes | No |

Abbreviations; SNPs, Single Nucleotide Polymorphisms; *ADAR1*, Adenosine Deaminase Acting on RNA; rSNP, rSNPBase identified regulatory SNPs; eQTL, Expression Quantitative Trait Loci.

**Supplementary table 2.** Summary of *in silico* functional predictions of rs2229857, rs1127313, rs1127314, rs1127317 and rs1127326 SNPs located within the *ADAR1* gene by using VEP tool (http://grch37.ensembl.org/info/docs/tools/vep/index.html).

|  | SNPs | Localization | Consequence | Amino-acids substituion | SIFT tool | PolyPhen tool |
| --- | --- | --- | --- | --- | --- | --- |
| VEP tool | rs2229857 | Exon 2 | Missense variant | K/R | Tolerated | Benign |
|  | rs1127313 | 3`UTR | 3`UTR variant | - | - | - |
|  | rs1127314 | 3`UTR | 3`UTR variant | - | - | - |
|  | rs1127317 | 3`UTR | 3`UTR variant | - | - | - |
|  | rs1127326 | 3`UTR | 3`UTR variant | - | - | - |

Abbreviations; SNPs, Single Nucleotide Polymorphisms; *ADAR1*, Adenosine Deaminase Acting on RNA; VEP, Ensembl’s Variant Effect Predictor; SIFT, Sorting Intolerant from Toleran; PolyPhen, Polymorphism Phenotyping.

**Supplementary table 3.** Summary of *in silico* functional predictions of rs2229857, rs1127313, rs1127314, rs1127317 and rs1127326 SNPs located within the *ADAR1* gene by using miRDB target prediction (<http://mirdb.org/miRDB/index.html>).

|  | **SNPs** | **miRNA binding site** |
| --- | --- | --- |
| miRDB target prediction | rs2229857 | - |
|  | rs1127313 | hsa-miR-4516 |
|  | rs1127314 | hsa-miR-363-5p |
|  | rs1127317 | - |
|  | rs1127326 | - |

Abbreviations; SNPs, Single Nucleotide Polymorphisms; *ADAR1*, Adenosine Deaminase Acting on RNA.

**Supplementary table 4.** Summary of *in silico* functional predictions of rs2229857, rs1127313, rs1127314, rs1127317 and rs1127326 SNPs located within the *ADAR1* gene by using F-SNP database (<http://compbio.cs.queensu.ca/F-SNP/>).

|  | SNPs | Splicing_regulation |
| --- | --- | --- |
| F-SNP | rs2229857 | Yes |
|  | rs1127313 | No |
|  | rs1127314 | No |
|  | rs1127317 | No |
|  | rs1127326 | No |

Abbreviations; SNPs, Single Nucleotide Polymorphisms; *ADAR1*, Adenosine Deaminase Acting on RNA.
